# The use of an avian macrophage-like cell line (HD11) transduced with recombinant lentiviruses to dissect the immunomodulatory role of Marek’s disease virus gene products

**DOI:** 10.1007/s11262-026-02224-z

**Published:** 2026-03-27

**Authors:** Sonsiray Alvarez-Narvaez, Steven J. Conrad, Taejoong Kim, Stephen Spatz, John R. Dunn

**Affiliations:** United States Department of Agriculture, Agricultural Research Service, US National Poultry Research Center, Athens, GA USA

**Keywords:** Marek’s disease virus, MDV, Mardivirus gallidalpha2, Recombinant lentivirus, Type I interferon, IFN-I, cGAS, STING, HD11

## Abstract

Marek’s disease (MD) is caused by the highly contagious Marek’s disease virus (MDV), a tumorigenic alphaherpesvirus. The disease is presently managed through vaccination. Nevertheless, the dynamic nature of MDV presents challenges, as new virulent strains may arise that can potentially overcome existing vaccine-mediated immunity. To address this, new and more effective vaccines are crucial. The immune system’s first line of defense against infections is the innate immune response, where type I interferons (IFN-I) play a key role. As with other alphaherpesviruses, MDV encodes numerous genes capable of modulating IFN-I production during infection. Ablation of these genes in MDV could result in attenuation, thereby facilitating the creation of novel vaccine strains with augmented IFN-I induction during infection. Hence, a system to identify immunomodulatory MDV gene products that impede IFN-I production in avian innate immune cells is required. Herein, we present an innovative screening method that quantifies the expression of Interferon Omega 1 (IFNω1) in an avian macrophage-like cell line expressing MDV genes via recombinant lentivirus (rLV) integration. Five MDV gene products hypothesized to inhibit IFN-I production (Meq, US3, R-LORF4, UL46, and UL48) were assayed in our screening system. Stable expression of all MDV genes was obtained when rLV was utilized to insert these genes into HD11 cells. Additionally, we established the optimal conditions for activation of the IFN-I response in HD11 cells using a 2 kb double-stranded DNA segment delivered by transfection as a stimulant. With this approach, we demonstrated that these MDV gene products significantly decreased the gene expression of IFN-I when activated by transfection with double-stranded DNA.

**Supplementary Information:**

The online version contains supplementary material available at 10.1007/s11262-026-02224-z.

## Introduction

Among poultry diseases, Marek’s disease (MD), a lymphoproliferative disease caused by the oncogenic alphaherpesvirus Marek’s disease virus (MDV), also known as Mardivirus gallidalpha2, is regularly listed as an issue affecting poultry producers [1]. Concern regarding MD is heightened due to the unpredictable yet recurrent vaccine breaks that have resulted in devastating losses to poultry farms [2, 3]. Estimated worldwide annual losses from MD due to condemnation and reduced egg production exceed $2 billion [4, 5]. As MDV is ubiquitous in the environment, the main MD control strategy is widespread vaccination of commercial flocks. The first MD vaccine used in the USA was herpesvirus of turkeys (HVT), a related turkey herpesvirus [6, 7]. Since then, more effective vaccines based on attenuated serotype 1 and naturally non-oncogenic serotype 2 MDV strains have been developed and used in various combinations [8]. It is important to note that while these vaccines are protective against disease development, they do not prevent MDV infection, viral replication, or viral shedding into the environment [9, 10]. Because MD-vaccinated birds survive the infection and shed the virus into the environment, MD vaccines may themselves drive the evolution of the virus to increasing levels of virulence [11–13], a problem which is exacerbated by high-density rearing conditions and shorter cohort lifetimes [14–16]. Thus, there is a need for new and more protective vaccines against the emergence of new and more virulent strains of MDV [17].

Several groups using a variety of virus types, including alphaherpesviruses such as feline herpesvirus 1 [18] (FHV-1), baboon papioine herpesvirus 2 [19] (HVP2), or even the human herpesvirus 1 [20] (HHV-1), have successfully attenuated virulent viruses through the ablation of virulence factors that act to inhibit IFN-I production. Type I IFNs are pleiotropic cytokines that act as autocrine, paracrine, and endocrine effector molecules and orchestrate the response to viral infections at the intracellular, intercellular, and organismal level [21]. The type I IFN production is a key early event in the protective response to viral infection. This is particularly relevant for herpesviruses that must inhibit the production of type I IFNs to establish a favorable environment for replication that ultimately leads to a successful infection [22–25]. Several MDV gene products have been reported to interfere with type I IFN production by antagonizing the cGAS–STING DNA-sensing pathway [26–29]. However, further research revealed that additional MDV virulence factors share significant homology with HHV-1 proteins known to inhibit type I IFN production. While Li et al. [30] used chicken embryo fibroblasts (CEFs) to rank MDV genes based on their ability to modulate the cGAS–STING pathway, we present a more biologically relevant system: an avian macrophage-like cell line (HD 11). Here, we describe a novel assay to identify MDV gene products that suppress IFN-I production by expressing them via recombinant lentiviruses (rLV) in the HD11 cell line.

## Materials and methods

### Cell culture

The chicken macrophage-like cell line HD11 [31] was generously donated by Professor Mark Parcells (University of Delaware). HD11 cells were routinely maintained at 38 °C with 5% CO_2_ in a maintenance medium that consisted of DMEM with 4.5-g/L D-glucose, L-glutamine, and 110-mg/L sodium pyruvate (Gibco catalog no. 11995-065) supplemented with 8% heat-inactivated fetal bovine serum ([HI-FBS], Biowest catalog no. S1480) and 0.01% gentamycin (Gibco catalog no. 15750-078).

### Activation of IFNω1 production in HD11 avian macrophages

HD11 cells were seeded into a 6-well plate (Corning, catalog no. 3335) at a concentration of 2 × 10^6^ cells/well and incubated at 38 °C with 5% CO_2_ overnight. On the following morning, the medium from each well was replaced with 1 mL of fresh, prewarmed maintenance medium. The transfection mix was prepared by adding 3 μL of TurboFectin 8.0 transfection reagent (Origene) for every 1 μg of double-stranded (ds) DNA to 250 μL of Opti-MEM medium (Thermo Fisher) per well. The mix was incubated at room temperature for 15 min and added to the cells dropwise. The control wells received Opti-MEM medium with an equal amount of TurboFectin 8.0 but no dsDNA. Plates were briefly rocked to ensure mixing and the plates were incubated between one and five hours (depending on the experiment) at 38 °C with 5% CO_2_. After that time, the cells were washed with 1 mL of prewarmed Dulbecco’s phosphate-buffered saline (DPBS) without calcium and magnesium and lysed with 350 μL of RLT buffer (RNeasy extraction kit, Qiagen catalog no. 74136) supplemented with 35 μL of 2-Mercaptoethanol (Corning, catalog no. 3008) before storage at −80 °C until further use.

### Conventional polymerase chain reaction

The dsDNA agonists of varying lengths used to stimulate the IFN pathway in HD11 cells were produced by polymerase chain reaction (PCR) amplification of several segments of the AIO-mCherry plasmid (Addgene catalog no. 74120) with a universal forward oligonucleotide (ampRuniv-F) and several reverse oligonucleotides (Table S1). The dsDNA amplicons were designed to lack open reading frames and promoters. The PCR master mix consisted of 25 μL of the 2 × GoTaq® Green Master Mix (Promega, catalog no. M7122), 5 μL of 10 μM forward primer (IDT), 5 μL of 10 μM reverse primer (IDT), 5 μL of plasmid DNA, and 10 μL of nuclease-free ultrapure water (Ambion, catalog no. AM9937). The PCR were run in a T100 Thermal Cycler (Bio-Rad) using the following conditions: an initial denaturation step for 3 min at 95 °C, followed by 40 amplification cycles (30 s at 95 °C of denaturation, 30 s of oligonucleotide hybridization at 63 °C, and 2 min of elongation at 72 °C), and a final elongation of 5 min at 72 °C. PCR products were electrophoresed (Figure S2) on a 1% UltraPure Agarose (Invitrogen, catalog no. 16500) in 1X TBE buffer (Promega, catalog no. V4251) gel, and visualized in an Alphaimager Model 2 (Alpha Innotech). Amplicon sizes were compared to a Promega BenchTop 100 bp DNA ladder (Promega catalog no. G8291). PCR products were purified using a QIAquick PCR purification kit (QIAGEN, catalog no. 28104).

### Determination of puromycin dosage in HD11 cells

HD11 cells were seeded at a density of 1 × 10^6^ cells per well in 12-well plates (Corning, catalog no. 3512) and incubated at 38 °C with 5% CO_2_ overnight. The following day, the medium from each well was replaced with 1.5 mL of fresh, prewarmed maintenance medium supplemented with 0.4, 0.8, 1.6, 3.2, or 4 μg/mL of puromycin (Gibco catalog no. A11138-03). At 0-h, 48-h and 120-h post-puromycin exposure, the cells were dissociated using 200 μL of TrypLE Express Enzyme (Gibco, catalog no. 12604021) at 38 °C with humidity and 5% CO_2_ for 10 min. TrypLE was neutralized with 800 μL of maintenance medium. Cell viability and count were assessed using Trypan Blue stain (Invitrogen catalog no. T10282) and Countess Cell Counting Chamber Slides (Invitrogen, catalog no. C10228) in a Countess 3 Automated Cell Counter (Invitrogen AMQAX2000) according to the manufacturer’s instructions.

### Lentivirus integration in the HD11 genome

rLV expressing MD genes were produced commercially (VectorBuilder Inc.). HD11 cells were infected with each rLV following the manufacturer’s instructions. Briefly, on the day before LV transduction (day 0), HD11 cells were plated at a density of 3 × 10^5^ cells/well in a 6-well plate (Corning catalog no. 3335) and incubated for 18–20 h at 38 °C in a humidified 5% CO_2_ incubator. On the day of transduction (day 1), the medium from each well was replaced with 1 mL of fresh, prewarmed maintenance medium containing the rLV at a concentration of 10^6^ virions/mL [multiplicity of infection (MOI) of 10]. The plate was gently swirled and incubated at 38 °C with humidity and 5% CO_2_ overnight. One day later (day 2), the virus-containing medium was replaced with fresh, prewarmed maintenance medium and the cells were again incubated at 38 °C in a humidified 5% CO_2_ incubator. When the transduced cells were confluent (day 4), they were dissociated from the 6-well plate using 200 μL of TrypLE Express Enzyme) and transferred to a T75 flask (Falcon, catalog no. 353133). Once the cells became confluent (day 7), the medium was exchanged for medium supplemented with 1.6 μg/mL of puromycin and incubated at 38 °C with humidity and 5% CO_2_ overnight. The following day, cells were washed twice with 1X DPBS, and fresh medium containing 3.2-µg/mL puromycin was added. Cells were incubated at 38 °C, 5% CO_2_, with regular puromycin replenishment (3.2 µg/mL) every 3 days and passaging as needed.

### Clonal selection of HD11 transduced cells

Puromycin-resistant HD11 cells in T75 flasks were rinsed with 1X DPBS and dissociated with 2 mL of TrypLE at 37 °C with humidity and 5% CO_2_ for 10 min. TrypLE was neutralized with 3 mL of medium supplemented with 3.2 μg/mL of puromycin. The cells were counted using Trypan Blue and Countess Cell Counting Chamber Slides in a Countess 3 Automated Cell Counter Cells were diluted in 96-well plates (Corning, catalog no. 3585) to achieve 1 cell/well in 200 μL of medium containing 3.2 μg/mL puromycin. The cells were grown for at least 10 days and inspected regularly for clonal colony formation in each well. Wells containing a single distinct colony were allowed to reach confluency, trypsinized with 20 µL of TrypLE, and transferred to a T25 flask (Falcon, catalog no. 353108) containing 5 mL of maintenance medium supplemented with 3.2 μg/mL puromycin. Upon reaching confluency, cells were further expanded into T75 flasks containing 12 mL of puromycin-supplemented maintenance medium. The integration of a control rLV expressing the eGFP marker was monitored on a BZ-X all-in-one fluorescence microscope (Keyence).

### RNA extraction and quantitative polymerase chain reaction

Total RNA was extracted using the RNeasy extraction kit (Qiagen, catalog no. 74136) following the manufacturer’s guidelines. cDNA was produced using the iScript cDNA Synthesis Kit (Bio-Rad, catalog no. 1708890). Each reaction used 200 ng of RNA, with 4 µL of 5 × iScript reaction mix, 1 µL iScript reverse transcriptase, and nuclease-free water for a final reaction volume of 20 µL as per the manufacturer’s instructions. Reactions were placed in a T100 Thermal Cycler (Bio-Rad) under the following conditions: 25 °C for 5 min; 46 °C for 20 min; 95 °C for 1 min; and 4 °C infinite hold. Quantitative PCRs (qPCRs) were carried out using the Luna Universal qPCR Master Mix (New England Biolabs, catalog no. M3003) in a QuantStudio 3 Real-Time PCR (Thermo Fisher Scientific). Samples were run in MicroAmp EnduraPlate Optical 96-well Clear Reaction plates with barcode (Thermo Fisher Scientific, catalog no. 4483354), and each 25 µL reaction contained 11.5 µL of the produced cDNA, 0.5 µL of forward primer (100 µM stock, 2), 0.5 µL of reverse primer (100 µM stock, 2 µM final concentration), and 12.5 µL of the 2X Luna Universal qPCR master mix. Thermal cycling parameters were set as follows: 95 °C for 5 min; 42 cycles of 95 °C for 15 s; and 63 °C for 1 min. Melt curves were constructed between 60 °C and 95 °C with a ramp increment of 0.3 °C. Melting curve data from each qPCR run was scrutinized to ensure all product amplicons displayed the correct melting point before inclusion in the analysis. Results were analyzed with the QuantStudio Design and Analysis software (v.1.5.3, Applied Biosystems). *GAPDH (*glyceraldehyde-3-phosphate dehydrogenase) (NCBI accession no. NM_204305.2) was used as a housekeeping gene for gene expression normalization. A table of oligonucleotide primers used in this study can be found in the supplementary materials (Table S1).

### Statistical analysis

The Wilcoxon signed-rank test was used to determine the significant differences in the IFNω1 expression fold change after stimulation with dsDNA. Multiple paired *t* tests were used to assess the significant differences in IFNω1 expression of stimulated HD11 cells expressing the different MDV gene products and the control eGFP gene. A threshold of *p* < 0.05 was used to determine statistical significance. All analyses were performed in statistical software GraphPad Prism 9.3.1 (La Jolla, USA).

## Results

### Optimizing the conditions for HD11 activation and IFN-I production

**Fig. 1:**
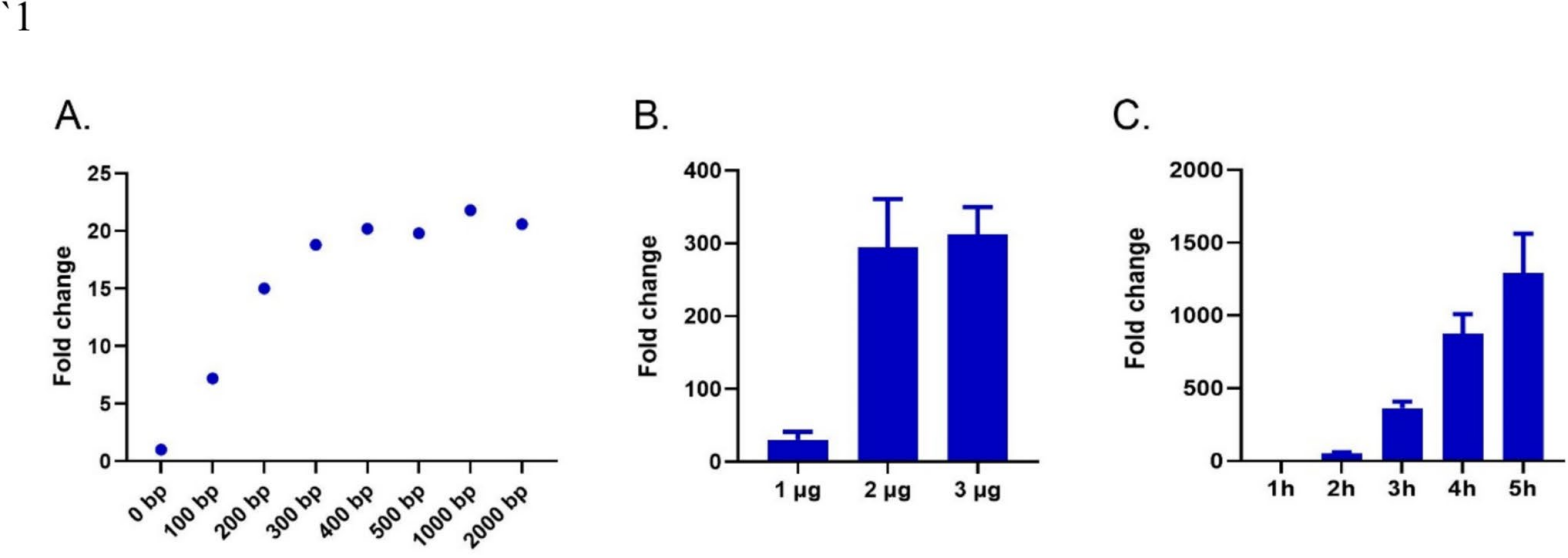
Optimizing the conditions for HD11 activation and IFN-I production. **A** Dot plot illustrating the *IFNω1* expression fold change over housekeeping gene *GAPDH* (Y-axis) in HD11 cells exposed to different sizes of dsDNA amplicons ranging between 100 and 2000 bp (X-axis). Cells were incubated with 1 µg of each amplicon for 3 h (*n* = 1 biological replicate). **B** Bar plot showing the *IFNω1* expression fold change over housekeeping gene *GAPDH* (Y-axis) in HD11 cells exposed to 1, 2, or 3 μg of 2 kb dsDNA (X-axis). Cells were incubated with the stimulants for 3 h (*n* = 3 biological replicates). **C** Bar plot showing the *IFNω1* expression fold change over housekeeping gene *GAPDH* (Y-axis) in HD11 cells exposed to 3 μg of 2 kb dsDNA for up to 5 h (X-axis) (*n* = 3 biological replicates)

To optimize macrophage stimulation for a robust and consistent IFN-I response, avian macrophage-like cells (HD11) were transfected with double-stranded DNA (dsDNA) amplicons of varying lengths (100–2000 base pairs) for 3 h. The fold change expression of the *IFNω1* gene (NCBI accession no. NM_001024836.2) relative to the housekeeping gene *GAPDH* (NCBI accession no. NM_204305.2) was used to quantify the effect of the stimulant dsDNA length on *IFNω1* expression (Fig. 1A).

We observed that the expression of *IFNω1* increased in a length-dependent manner and plateaued when PCR amplicons with lengths over 500 bp were used. For all future experiments, a 2-kb dsDNA amplicon was used as the stimulant. Next, the optimum amount of the 2-kb dsDNA stimulant for *IFNω1* production was determined. HD11 cells were transfected with increasing quantities (1 μg, 2 μg, and 3 μg) of the 2 kb amplicon for 3 h (Fig. 1B). The highest and more consistent *IFNω1* expression was obtained when using 3 μg (312.5 ± 37.5 [Mean ± S.E.M] fold increase over *GAPDH*) compared to the 294.2 ± 66.5- and 30.0 ± 11.2-fold change obtained with 2 μg and 1 μg, respectively. Henceforth, 3 μg of 2-kb dsDNA stimulant was used to stimulate HD11 cells in our experiments. Finally, a time course experiment was performed to determine maximal *IFNω1* expression using 3 µg of 2 kb amplicons (Fig. 1C). *IFNω1* expression plateaued after four hours of continuous exposure to the stimulant (877.2 ± 132.6 [Mean ± S.E.M] fold increase over *GAPDH*), with the highest *IFNω1* expression obtained after a 5-h incubation (1290.3 ± 271.7 [Mean ± S.E.M] fold increase over *GAPDH*). Overall, these experiments determined that a 5-h continuous exposure to 3 μg of a 2-kb dsDNA amplicon was optimal for inducing a robust and consistent IFN-I response in HD11 cells.

### Stable expression of MDV genes in HD11 chicken macrophage cell line

**Fig. 2:**
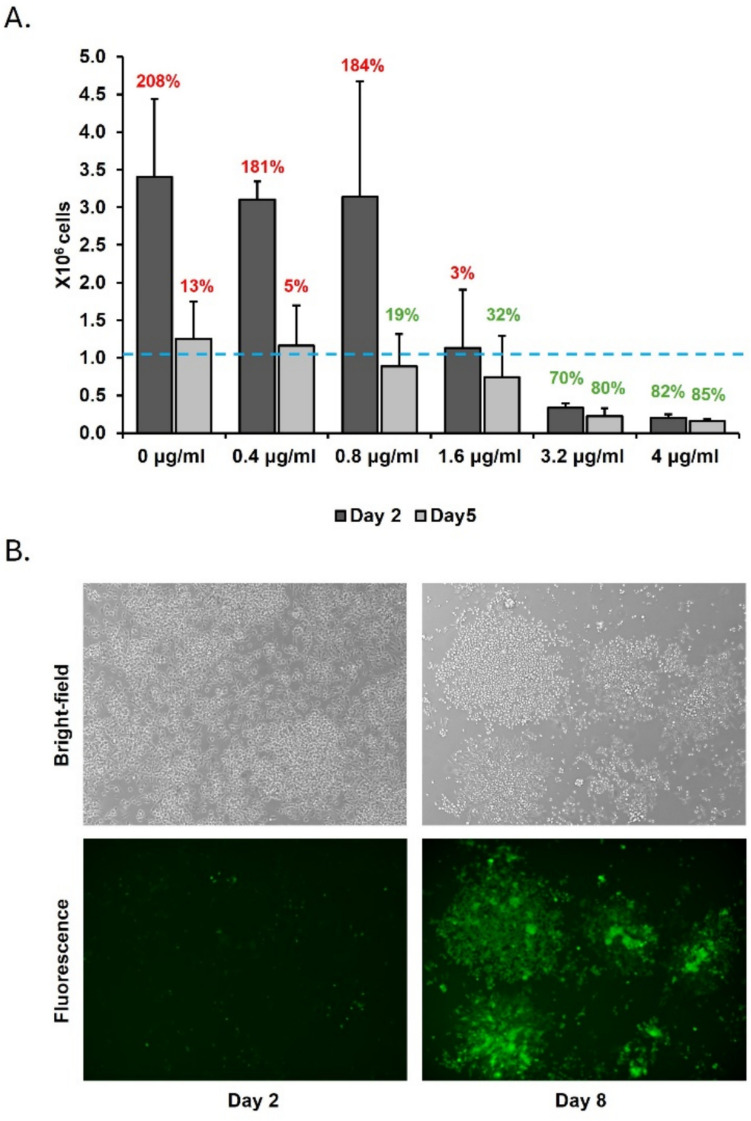
Establishing the optimal puromycin concentration for the selection of lentivirus-transduced cells. A. Bar plot showing the number of viable cells left (Y-axis) after a two- (dark gray) or five- (light gray) day incubation in the presence of different concentrations of puromycin (X-axis). The dashed line indicates the average number of cells at day 0. The numbers illustrate the percentage increase (red) or decrease (green) from the initial cell counts (n = 3 biological replicates). B. eGFP expression in HD11 2 and 8 days after infection with recombinant lentivirus lenti_GFP. Photos were taken in a Keyence BZ-X all-in-one fluorescence microscope under bright-field (top panels) and fluorescent light (bottom panels)

**Fig. 3:**
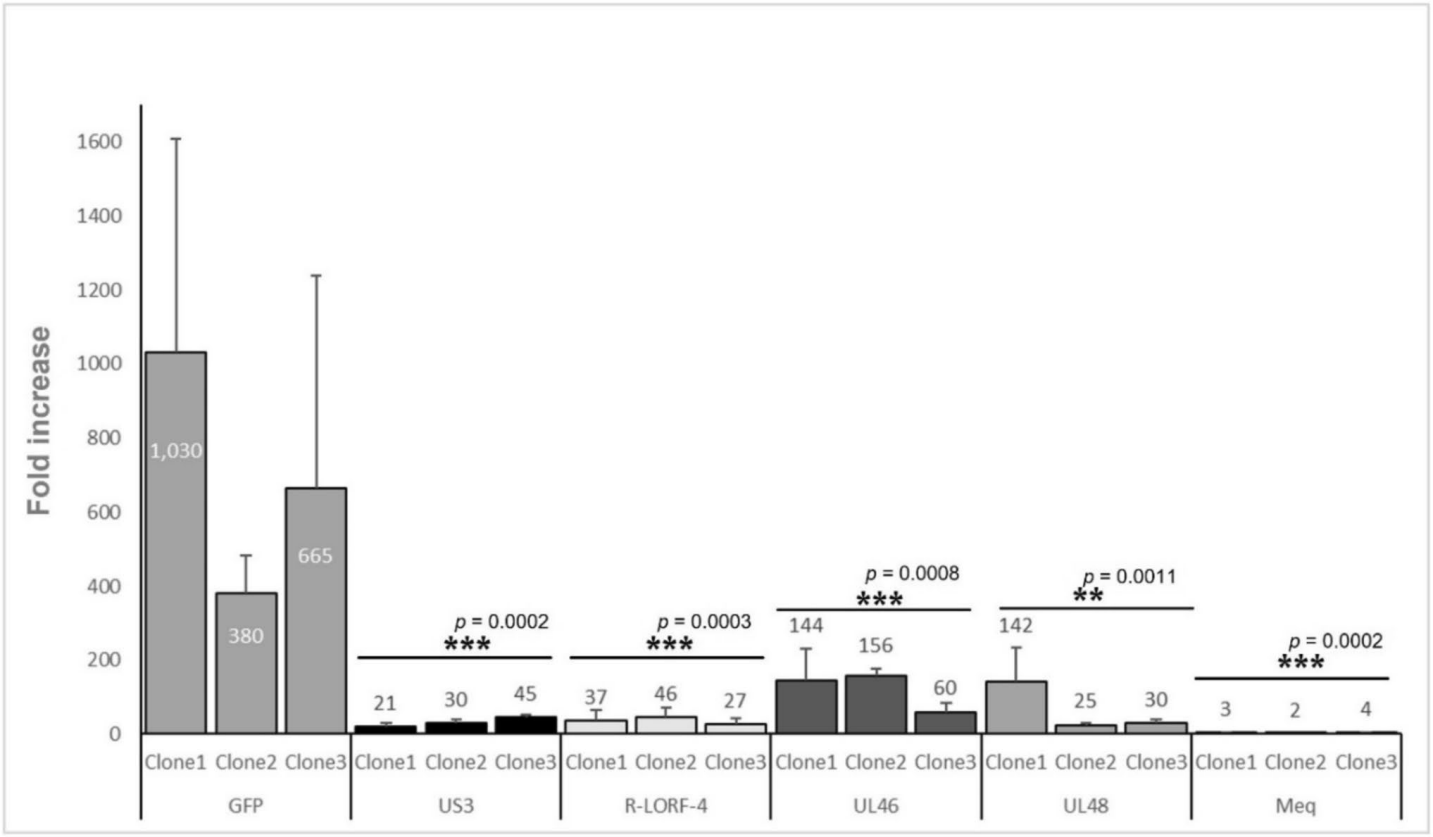
Bar-graph illustrating *IFNω1* gene expression fold increase over housekeeping gene *GAPDH* (Y-axis) in HD11 cells producing MDV gene products US3, R-LORF4, UL46, UL48, and Meq, and fluorescence tag eGFP (X-axis) via the integration and expression of six recombinant lentiviruses. *IFNω1* and *GAPDH* gene expression were measured in three clonal cell populations (clone 1–3) after their activation with 3 μg of 2-kb dsDNA for 5 h. Multiple paired *t* tests were used to assess the significant differences in the IFNβ expression of activated HD11 producing the different MDV gene products and the control eGFP gene. A threshold of *p* < 0.05 was used to determine statistical significance (*n* = 3 biological replicates per clone)

With an optimized assay system to measure IFN-I production in avian cells established, the expression of MDV genes in HD11 cells transduced with rLV was assessed using real-time qPCR. Seven rLV, each carrying one of the MDV strain MD5 (NCBI accession number AF243438) genes *Meq*, *US3*, *R-LORF4*, *UL18, UL46, UL48*, or the *eGFP* gene, were used to infect HD11 cells. Expression of each transgene was driven by the human cytomegalovirus (CMV) immediate early promoter. Puromycin resistance (via a puromycin resistance cassette also driven by the CMV promoter) was used to select successfully transduced cells. A schematic representation of one of the plasmids used to construct the lentiviral vectors in *E. coli* can be found in the supplementary materials (Figure S3). To determine the intrinsic resistance of HD11 cells to puromycin, unaltered (wild type) HD11 cells were incubated in the presence of increasing concentrations of puromycin (0–4 μg/mL) for up to 5 days, and cell viability was measured at 0, 2, and 5 days after puromycin introduction (Fig. 2A). The cell appearance in the wells under the lowest puromycin concentrations 0.4 μg/mL and 0.8 μg/mL was comparable to the untreated cells, indicating that these two concentrations did not affect HD11 viability. However, in these groups, a decrease in cell viability was observed at day 5, most probably caused by cell overgrowth as indicated by acidification of the cell culture medium and corresponding color change (from bright pink to yellow). Two days after the application of 1.6 μg/mL of puromycin, the cells reached an equilibrium between cell replication and death, with a decrease in cell viability (32%) after 5 days of treatment. A consistent decrease in cell viability over time was observed in cells treated with higher concentrations (3.2 and 4 μg/mL). The application of 3.2-μg/mL puromycin concentration more gradually reduced HD11 cell viability in a more staggered manner compared to 4 μg/mL, leading us to choose the former for subsequent experiments. To validate this selection, HD11 cells were transduced with a control lentiviral vector expressing eGFP, and an increasing number of fluorescent cells over time was observed, confirming successful selection of transduced cells (Fig. 2B). Due to the random nature of rLV integration and its potential impact on transgene expression, we generated multiple clonal lines for further analysis. Three clones per construction were selected and verified for eGFP expression using fluorescence microscopy and qPCR (Fig. 3).

### Immunomodulatory effects of MDV gene products in the production of IFN-I in stimulated HD11 cells

Finally, we used our newly developed assay to screen the MDV gene products Meq, US3, R-LORF4, UL46, and UL48 for their ability to inhibit the production of IFNω1 after stimulating HD11 cells with dsDNA. As was done previously for the eGFP-carrying HD11 clone, three clonal populations were generated for each gene. On average, the control (eGFP only) cell line exhibited an approximately 600-fold increase in IFNω1 expression when stimulated by dsDNA under our standard assay conditions (Fig. 3). These IFNω1 expression levels are in agreement with our observations during our assay optimization experiments (Fig. 1), indicating that neither the insertion of the recombinant LV (and the resulting puromycin resistance) in the HD11 genome, nor the production of eGFP interfere with *IFNω1* gene expression. On the other hand, all the MDV gene products tested significantly impacted the expression of IFNω1 mRNA (Fig. 3). The presence of Meq in the activated HD11 cells completely obliterated the expression of IFNω1 to an average of only a threefold increase. US3 and R-LORF4 similarly reduced IFNω1 expression to a 32- and 38-fold increase. On the lower end, but still highly significant, is the effect of UL48 and UL46, which reduced the expression of IFNω1 to an average of 66- and 120-fold increase, respectively (Fig. 3). To demonstrate that the rLV transduction of HD11 did indeed result in the production of the intended gene product, we verified the production of *meq* mRNA, whose gene product shows the highest inhibitory effect in our assay, by qPCR in all of the rLV meq-transduced clones (Table S4). Overall, these results demonstrate that we have successfully developed a method to assess the ability of individual viral genes to contribute to the virus’s ability to frustrate the IFN-I response in a biologically relevant system, such as the avian macrophage.

## Discussion

The ablation of viral genes encoding virulence factors that inhibit IFN-I response is a proven strategy for viral attenuation [32] that could be used to create new MDV vaccine strains with improved protection. Herein, we detail our efforts to develop and optimize an assay for the identification of MDV genes that modulate the host production of IFN-I and demonstrate that the MDV gene products Meq, US3, R-LORF4, UL48, and UL46 significantly reduce the amount of IFNω1 produced when stimulated by transfected dsDNA.

The cGAS–STING DNA-sensing pathway is pivotal in inducing the IFNω1 response against MDV infection in chicken [26], although other cellular sensors such as DAI [33] and AIM2 [34] certainly contribute to some extent and may also exert their effects through the cGAS–STING transduction cascade [35]. The cGAS (GMP-AMP synthase) is a cytosolic sensor [36] that interacts with several forms of nucleic acids, including ssDNA [37], dsDNA [38], and DNA-RNA hybrids [39]. As MDV is a dsDNA virus [40], and degradation of viral capsids causes the release of genomic dsDNA into the cytoplasm [41], we decided to simulate infection and induce the activation of the cGAS–STING pathway in HD11 using dsDNA as stimulant. In mammals, cGAS has been shown to respond to cytoplasmic dsDNA in a length-dependent manner [42, 43], but its length dependency has not been examined in avians. To determine the optimum length of dsDNA to induce the production of IFNω1 in HD11 cells, we transfected various lengths of dsDNA into the cells and showed that the production of IFN-I is activated in a length-dependent manner as well, with maximal stimulation achieved at approximately 500 bp and above. This leads us to believe that the IFN-I stimulation of HD11 in this study is indeed mediated by the cGAS–STING axis, but we do not rule out the involvement of other cytosolic dsDNA sensors. A study using human and murine cell lines reported that robust IFNβ expression (200-fold increase upon stimulation) was achieved by exposing the cells to 2 μg of dsDNA for 6 h [44, 45]. Similar findings were obtained in the HD11 avian cell line that reached maximum IFNω1 expression when using > 2 μg and after five hours of exposure. Taken together, these data suggest that the production of IFNω1 shares similar timing and length dependency in both mammalian and avian cells.

Lentiviral vectors can deliver and express genes in a wide variety of dividing and nondividing cells by integrating their genetic material into the host cell’s genome [46]. We decided to explore the usage of rLV for a constitutive expression of MDV genes in HD11 because lentiviruses can package large amounts of foreign DNA (~ 10 kb) with prolonged transgene expression [47–50]. Our commercially produced rLVs were pseudotyped with the envelope protein of the vesicular stomatitis virus, the VSV glycoprotein G, to enhance gene transfer into multiple hematopoietic cell types [51]. The MDV genes were expressed in HD11 cells under the control of the human cytomegalovirus (HCMV), a strong constitutive promoter for robust gene expression [52, 53]. Transduced cells were selected with puromycin as previously described. [54–56]. As anticipated, our results demonstrate robust expression of the integrated MDV *meq* gene inserted and the fluorescence GFP tag, validating the efficacy of both our gene delivery and expression system, as well as our puromycin selection protocol. With clonally selected HD11 cell lines that consistently expressed MDV genes and the optimized assay conditions for HD11 stimulation, we proceeded to investigate the impact of MDV proteins on IFN-I production. Recently, Li et al. systemically evaluated the impact of individual MDV gene products on their ability to modulate the cGAS–STING pathway [30]. Using an immortalized chicken fibroblast cell line (DF-1), the production of IFNω1 was measured following cotransfection of a luciferase reporter under the control of an IFNω1-inducible promoter and plasmids expressing cGAS and STING [30]. To identify the MDV genes encoding the most IFN-suppressive proteins, we consulted the findings of Li et al., who ranked the MDV gene products based on their ability to modulate the cGAS–STING pathway and selected the top ten most impactful genes for further evaluation in our system.

In the classification by Li et al., Meq was the most inhibitory gene product, followed by R-LORF4, US3, and UL46. UL48 was ranked number 11. For some of these proteins, the molecular mechanisms related to the regulation of the cGAS–STING pathway are known [28]. For instance, Meq (the oncoprotein of MDV [57]) has been recently shown to inhibit the action of STING by impairing the assembly of the STING–TBK1–IRF7 complex [30]. Similarly, there is evidence that the multifunctional kinase US3 [58] disrupts the association between TBK1 and IRF7 by phosphorylating IRF7 and blocking its nuclear translocation. On the other hand, ectopically expressed R-LORF4 has also been observed to block IFN-β promoter activation but by selectively inhibiting the activation of the nuclear factor kappa-B (NF-κB) [27], so this gene product intervenes in the IFNω1 production cascade later than at the dsDNA-sensing step. The specific role of the viral tegument proteins UL46 and UL48 [59] in the modulation of the IFN-I production in the host is still unknown. Our experimental results confirmed the inhibitory effects of all tested MDV genes on IFNω1 production, aligning with the findings of Li et al. [30]. Notably, our assay further validated the hierarchical impact of these genes, with Meq emerging as the primary IFN-I inhibitor, followed by US3, R-LORF4, UL48, and UL46, in the context of a highly relevant biological system, avian macrophages.

## Supplementary Information

Below is the link to the electronic supplementary material.

Supplementary file1 (DOCX 155 KB)

## Data Availability

No datasets were generated or analyzed during the current study.
