# Supplementary material for: The use of an avian macrophage-like cell line (HD11) transduced with recombinant lentiviruses to dissect the immunomodulatory role of Marek’s disease virus gene products
Source: Virus Genes. 2026 Mar 27;62(3):318–27. doi: 10.1007/s11262-026-02224-z (PMC13226417; doi:10.1007/s11262-026-02224-z)
Supplement: Supplementary file 1 — Supplementary file1 (DOCX 155 KB) [file 11262_2026_2224_MOESM1_ESM.docx]

Table S1. List of oligos used in this study

| **Name** | **Sequence [5'-3']** | **Amplicon size [bp]** | **Description** |
| --- | --- | --- | --- |
| ampRuniv_F | CCGTGTCGCCCTTATTCCC | - | Universal forward primers to produce all dsDNA amplicons |
| ampR100_R | ACCCAACTGATCTTCAGCATC | 100 | Reverse primer to produce a dsDNA 100bp amplicon |
| ampR200_R | GAACTTTAAAAGTGCTCATCATTGG | 200 | Reverse primer to produce a dsDNA 200bp amplicon |
| ampR300_R | ACTGGTGAGTACTCAACCAAGTC | 300 | Reverse primer to produce a dsDNA 300bp amplicon |
| ampR500_R | TTTGGTATGGCTTCATTCAGCTC | 500 | Reverse primer to produce a dsDNA 500bp amplicon |
| ampR1000_R | CTTTTCTACGGGGTCTGACGC | 1000 | Reverse primer to produce a dsDNA 1000bp amplicon |
| ampR2000_R | CTCGGTGCCACTTTTTCAAGTTG | 2000 | Reverse primer to produce a dsDNA 2000bp amplicon |
| GAPDH_F5 | CTGAATGGGAAGCTTACTGGAATG | 222 | Primers used to measure the expression of *GAPDH* gene |
| GAPDH_R5 | CGCATCAAAGGTGGAGGAATG |  |  |
| IFNW1_F4 | GCTCACCTCAGCATCAACAAATAC | 445 | Primers used to measure the expression of *IFNW1* gene |
| IFNW1_R4 | GGCAAAGACTTCCTCTTTCCATTC |  |  |
| Meq_F4 | AGGGCCAATGAACACCTACG | 181 | Primers used to measure the expression of *meq* gene |
| Meq_R4 | GTGGAGGAGTGCAAATGGGA |  |  |


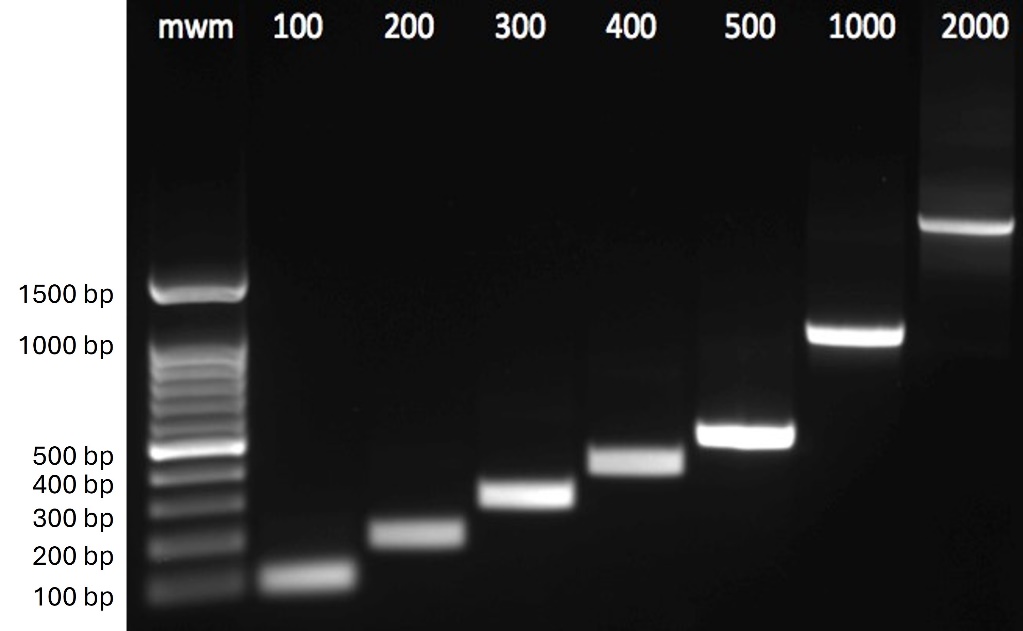


Figure S2. Visualization of the dsDNA amplicons used to activate HD-11. The mwm column includes the Promega BenchTop 100bp DNA ladder. Numbers on top of each column correspond to the size of each expected amplicon.


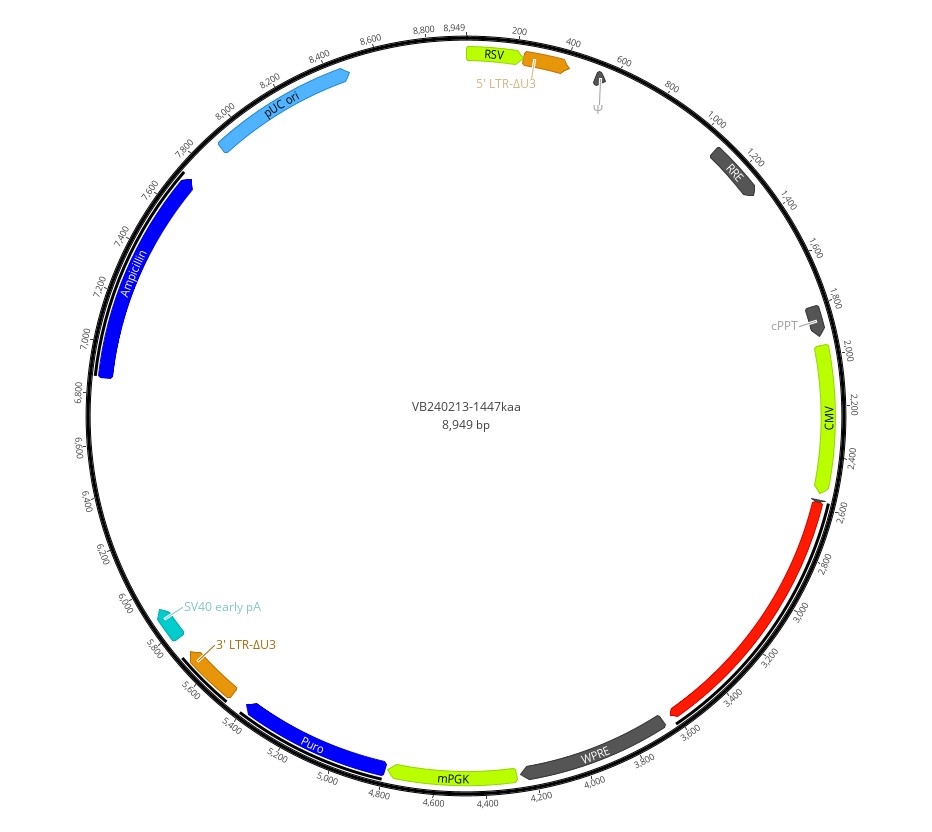
Figure S3. Schematic representation of one of the plasmids used to construct the lentiviral vectors in *E. coli.* Green arrows represent promoters CMV (human cytomegalovirus immediate early enhancer/promoter), mPGK (mouse phosphoglycerate kinase 1 promoter) and RSV (rous sarcoma virus enhancer/promoter). Blue arrows represent puromycin and ampicillin resistant cassettes. Grey arrows represent miscellaneous factors that that help in lentivirus packaging such as genes WPRE (Woodchuck hepatitis virus posttranscriptional regulatory element) and RRE (HIV-1 Rev response element), Ψ HIV-1 packaging signal and cPPT (central polypurine tract). The orange arrows represent the Truncated HIV-1 3' (3' LTR-ΔU3) and the 5' (5' LTR-ΔU3) long terminal repeats. The light blue and the cyan arrows represent the pUC origin of replication (pUC ori) and the simian virus 40 early polyadenylation signal (SV40 early pA) respectively. In red the MDV gene of interest.

Table 4. *meq* and *gapdh* gene expression levels in HD11 clonal populations measured by qPCR. Gene expression was assessed in the three independent experiments performed for each clonal population. All samples were tested in quadruplicate, and results are presented as the mean ± SEM.

| **HD11 clonal population** | **Experiment** | ***GAPDH* C_T_** | ***Meq* C_T_** |
| --- | --- | --- | --- |
| 1 | 1 | 14.00±.04 | 20.00±.01 |
|  | 2 | 14.39±.05 | 21.95±.03 |
|  | 3 | 14.74±.05 | 21.93±.01 |
| 2 | 1 | 13.84±.05 | 19.02±.01 |
|  | 2 | 13.47±.02 | 20.34±.02 |
|  | 3 | 14.04±.04 | 20.39±.02 |
| 3 | 1 | 14.10±.03 | 19.40±.02 |
|  | 2 | 14.06±.03 | 20.71±.02 |
|  | 3 | 13.96±.03 | 21.62±.01 |
